# Low Lymphocyte Count Is Associated With Radiotherapy Parameters and Affects the Outcomes of Esophageal Squamous Cell Carcinoma Patients

**DOI:** 10.3389/fonc.2020.00997

**Published:** 2020-06-23

**Authors:** Xin Wang, Zongxing Zhao, Peiliang Wang, Xiaotao Geng, Liqiong Zhu, Minghuan Li

**Affiliations:** ^1^Department of Clinical Medicine, Shandong First Medical University and Shandong Academy of Medical Sciences, Jinan, China; ^2^Department of Radiation Oncology, Shandong Cancer Hospital and Institute, Shandong First Medical University and Shandong Academy of Medical Sciences, Jinan, China; ^3^Department of Radiation Oncology, Liaocheng People's Hospital, Liaocheng, China; ^4^School of Medicine, Shandong University, Jinan, China

**Keywords:** radiotherapy, esophageal squamous cell carcinoma, radiation-induced lymphopenia, prognosis, immunosuppression

## Abstract

**Purpose:** Lymphocytes are central players in systemic anti-tumor immune responses. In this study, we aimed to identify the relationship between absolute lymphocyte count (ALC) nadir during definitive radiotherapy (RT) and survival outcomes in patients with esophageal squamous cell carcinoma (ESCC), as well as evaluate the effect of RT parameters on ALC during RT.

**Materials and methods:** We retrospectively reviewed 189 patients with stage I-IVA ESCC, who were treated with definitive RT at a single institution between 2012 and 2015. ALC values were assessed before, weekly during RT, and 1 month after the end of RT. Kaplan–Meier and Cox regression analyses were used to evaluate the relationship between ALC nadir during RT and patient outcomes. Predictors of low ALC nadir were assessed using univariate and multivariate logistic regression analyses.

**Results:** The median ALC before treatment was 1.73 × 10^3^ cells/μL. Fifty-eight (58.2) percent of the patients exhibited low ALC nadir (≤ 0.38 × 10^3^ cells/μL) during RT. A low ALC nadir during RT was significantly associated with poor OS, PFS, and LRFS. The planning target volume (PTV) was larger in patients with low ALC nadir compared with patients with high ALC nadir (418.5 vs. 347.7 cm^3^, *P* = 0.023). Multivariate logistic regression analysis revealed that tumor stage III-IVA (*P* = 0.002), low ALC before treatment (*P* = 0.028), large Log_10_(PTV) (*P* = 0.01), high heart V10 (*P* = 0.003), and high heart V20 (*P* = 0.028) were associated with low ALC nadir during RT.

**Conclusions:** In ESCC patients who received definitive RT, a low ALC nadir during RT was associated with large PTVs, and it was an independent prognostic factor of outcomes.

## Introduction

Esophageal cancer (EC) is one of the most aggressive malignancies (1). Squamous cell carcinoma is the predominant histological type of EC in China, accounting for more than 90% of the total number of EC cases (2). Definitive chemoradiotherapy (dCRT) is the treatment of choice for unresectable or inoperable EC (3, 4). However, radiotherapy (RT) has various effects on the immune system of patients. Importantly, lymphocytes are the most radiosensitive cells of the hematopoietic system and are frequently depleted after RT, even at low radiation doses; hence, radiation-induced lymphopenia (RIL) is a common adverse effect of RT (5, 6).

Mounting evidence indicates that the immune system has multiple mechanisms for the identification and elimination of tumor cells, and lymphocytes are key players in these processes (7, 8). Several studies have demonstrated that RIL is associated with worse outcomes in a wide variety of malignancies, including EC (9–14). Moreover, it has been shown that in patients with stage I-III EC receiving chemoradiotherapy (CRT) with or without surgery, RIL is a significant prognostic factor associated with inferior survival (13, 14). Nevertheless, in the majority of previous studies, the most common pathological type (more than 80% of the cases) was esophageal adenocarcinoma (EAC). Moreover, the prognostic value of absolute lymphocyte count (ALC) in patients with esophageal squamous cell carcinoma (ESCC) treated with definitive RT remains elusive. In this study, we sought to identify the relationship between ALC during definitive RT and survival outcomes in ESCC patients, as well as to evaluate the effect of RT parameters on ALC.

## Materials and Methods

### Patient Selection

We retrospectively reviewed the records of ESCC patients who underwent definitive RT at a single institution from 2012 to 2015. Clinical TNM staging in all patients was performed according to the 8th edition of the American Joint Committee on Cancer (AJCC) guidelines. Study inclusion criteria were as follows: (1) Histologically or cytologically confirmed, treatment-naive esophageal squamous cell carcinoma; (2) stage I–IVA disease as determined by either contrast-enhanced computed tomography (CT), endoscopic ultrasonography (EUS) or positron emission tomography (PET)-CT; (3) Eastern Cooperative Oncology Group (ECOG) performance status 0–2; (4) patients with complete hematological data; (5) patients that completed the definitive RT plan. Patients with prior RT, second primary tumors or comorbidities that might have affected the lymphocyte count (e.g., autoimmune or inflammatory disorders) were excluded from the study.

### Treatment Protocols

All the patients of this study received definitive RT alone or in combination with chemotherapy. All radiation treatments were delivered either as three-dimensional conformal radiotherapy (3D-CRT) or intensity modulated radiotherapy (IMRT) with a total dose of 50–68 Gy. Radiation was delivered by high-energy (6 or 15 MV) linear accelerators. According to the chemotherapy combination regimens, patients were divided into the following subgroups: RT alone, induced chemotherapy, concurrent CRT, and induced chemotherapy + concurrent CRT. In patients that underwent concurrent CRT, chemotherapy began on day 1 concurrent with the initial radiation treatments session; the concurrent CRT regimen was platinum and 5-fluorouracil (5-FU) doublet chemotherapy, a combination of platinum and taxane, or other commonly used chemotherapeutic agents.

### Data Collection

ALC values were obtained within 1 week prior to treatment initiation, weekly during RT, and 1 month after RT. For patients with missing ALC data at a time point of interest, the closest value to the desired date was used. ALC nadir was defined as the minimum ALC value measured during RT. Lung dose-volume histogram (DVH) parameters, heart DVH parameters, and planning target volume (PTV) during RT were collected. Evaluation of the response of the primary tumor to treatment was performed using CT and esophagography 4–6 weeks after the completion of the treatment; response was defined according to the Response Evaluation Criteria in Solid Tumors (RECIST) guideline version 1.1 (15).

### Statistical Analysis

All statistical analyses were performed using SPSS version 22.0 (SPSS, Chicago, IL, USA) software package. Descriptive statistics were used to summarize patient characteristics at baseline, while lymphocyte nadir values over time were plotted to visualize the cell counts trends during treatment. Receiver operating characteristic (ROC) curve analysis was used to determine the optimal cut-off value, with ALC nadir during RT and complete response (CR) rate as test and state variables, respectively. The relationship between ALC nadir (high vs. low) and the CR rate was assessed using Pearson's chi-squared test. The primary endpoints of the study were overall survival (OS), progression-free survival (PFS), and local recurrence-free survival (LRFS), all of which were determined from the day of therapy initiation until an event or censor. Survival curves were generated using the Kaplan-Meier method, and survival outcomes according to ALC nadir during RT were compared using the log-rank test. Univariate and multivariate analyses were conducted using the Cox proportional hazards model to determine risk factors for survival. Covariates identified by univariate analysis (UVA) with *P*-value < 0.10 were incorporated in the multivariate model, which was constructed with the forward stepwise method. Patient and treatment characteristics in each group (high ALC nadir vs. low ALC nadir) were tested for associations using Pearson's chi-squared test. Logistic regression analysis was used to identify RT-related factors that are associated with low ALC nadir during RT. Stepwise multivariate logistic regression analyses were used to assess the variables with *P*-value < 0.10 in the UVA. Pearson correlation coefficients (r) were used to determine the association between ALC nadirs and log_10_ (PTV). All statistical tests were two-sided, and *P*-value < 0.05 was used to indicate statistical significance.

## Results

### Patient Characteristics

A total of 189 ESCC patients were included in this study. The median age at diagnosis was 67 years (range, 44–92 years), and male patients accounted for 74.6% of the study population. The majority of patients (72%) had stage III disease. The median primary tumor length was 4.4 cm (range, 0.9–17 cm). Most patients (52.4%) received RT combined with chemotherapy, 39.2% of which underwent concurrent CRT, and 24.3% underwent induced chemotherapy. The majority of patients that underwent concurrent chemotherapy received platinum and 5-FU doublet chemotherapy (16.9%), followed by the combination of platinum and taxane (12.7%). A higher portion of patients underwent IMRT (65.1%) than 3D-CRT (34.9%). The median follow-up time was 46 months. Additional information about patient demographics, tumor characteristics, and treatment regimens are listed in Table 1.

**Table 1:** Baseline patient, tumor, and treatment characteristics.

**Characteristic**	**All patients *N =* 189**	**High ALC nadir *N =* 79**	**Low ALC nadir *N =* 110**	***p*-value**
Age, n (%)				0.797
≤67 years	96 (50.8)	41 (42.7)	55 (57.3)	
>67 years	93 (49.2)	38 (40.9)	55 (59.1)	
Sex, n (%)				**0.044**
Male	141 (74.6)	53 (37.6)	88 (62.4)	
Female	48 (25.4)	26 (54.2)	22 (45.8)	
Smoking, n (%)				0.206
Ever	94 (49.7)	35 (37.2)	59 (62.8)	
Never	95(50.3)	44 (46.3)	51 (53.7)	
Drinking, n (%)				0.339
Ever	77 (40.7)	29 (37.7)	48 (62.3)	
Never	112 (59.3)	50 (44.6)	62 (55.4)	
Tumor location, n (%)				0.147
Cervical	17 (9.0)	4 (23.5)	13 (76.5)	
Upper	76 (40.2)	37 (48.7)	39 (51.3)	
Middle	51 (27.0)	23 (45.1)	28 (54.9)	
Lower	45 (23.8)	15 (33.3)	30 (66.7)	
Tumor length (cm)				**0.006**
≤4.4 cm	95 (50.3)	49 (51.6)	46 (48.4)	
>4.4 cm	94 (49.7)	30 (31.9)	64 (68.1)	
Clinical stage, n (%)				**0.002**
I	21 (11.1)	13 (61.9)	8 (38.1)	
II	20 (10.6)	14 (70.0)	6 (30.0)	
III	136 (72.0)	50 (36.8)	86 (63.2)	
IVA	12 (6.3)	2 (16.7)	10 (83.3)	
Treatment regimen, n (%)				0.910
RT combined with chemo	99 (52.4)	41 (41.4)	58 (58.6)	
RT alone	90 (47.6)	38 (42.2)	52 (57.8)	
Concurrent chemo regimen, n (%)				0.158
1. Platinum/taxane	24 (12.7)	10 (43.5)	13 (56.5)	
2. Platinum/5-FU	32 (16.9)	8 (25.0)	24 (75.0)	
3. Other	18 (9.5)	9 (50.0)	9 (50.0)	
RT technology, n (%)				0.172
3D-CRT	66 (34.9)	32 (48.5)	34 (51.5)	
IMR	123 (65.1)	47 (38.2)	76 (61.8)	
RT dose, n (%)				0.476
≤60 Gy	146 (77.2)	59 (40.4)	87 (59.6)	
>60 Gy	43 (22.8)	20 (46.5)	23 (53.5)	
Pre-treatment ALC, n (%)				0.180
≤1.73 × 10^3^ cells/μL	97 (51.3)	36 (37.1)	61 (68.9)	
>1.73 × 10^3^ cells/μL	92 (48.6)	43 (46.7)	49 (53.3)	

*ALC, absolute lymphocyte count; RT, radiotherapy; chemo, chemotherapy; 5-FU, 5-fluorouracil; 3D-CRT, three-dimensional conformal radiotherapy; IMRT, intensity modulated radiotherapy. Bold values indicate a statistically difference in statistical analysis (P < 0.05)*.

### Changes in Lymphocyte Count Over Time

**Figure 1:**
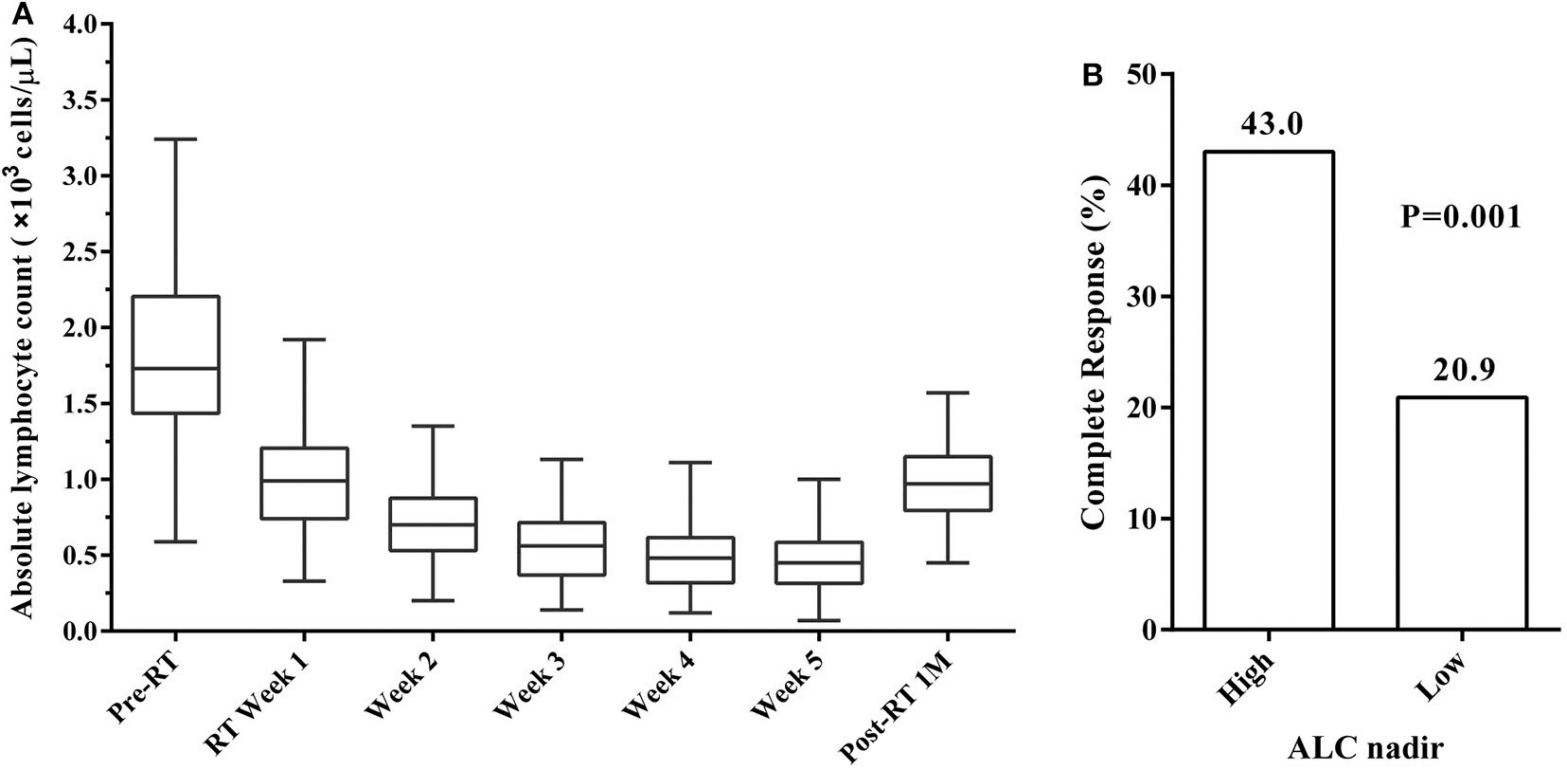
**(A)** Absolute lymphocyte count (ALC) trend from before radiotherapy (Pre-RT) through to 1 month after radiotherapy (Post-RT 1M). **(B)** Complete response (CR) rates by ALC nadir. CR rates was significantly higher in patients with high ALC nadir compared to those with low ALC nadir.

To get more insight into the changes in ALC during RT, ALC values were assessed over time (Figure 1A). The median ALC before treatment was 1.73 × 10^3^ cells/μL. As expected, ALC decreased every week during RT, reaching a plateau by the end of RT in most patients. The ALC values during RT decreased to 0.99, 0.70, 0.56, 0.48, and 0.45 × 10^3^ cells/μL from weeks 1 to 5, respectively. After the end of RT, ALC began to enter a slow recovery stage and returned to 0.97 × 10^3^ cells/μL by 1 month post-RT.

ROC curve analysis was performed to investigate the predictive value of ALC nadir during RT in CR rates. The optimal cut-off value of ALC nadir was determined as 0.38 × 10^3^ cells/μL, and the sensitivity and specificity were 63.2 and 63.6%, respectively. We, therefore, defined ALC nadir >0.38 × 10^3^ cells/μL as “high” and ≤0.38 × 10^3^ cells/μL as “low” ALC nadir. High ALC nadir was significantly associated with higher CR rates (Pearson's chi-square test; *P* = 0.001; Figure 1B). In the 189 patients, the median ALC nadir during RT was 0.34 × 10^3^ cells/μL (range, 0.07–1 × 10^3^ cells/μL). 79 (41.8%) patients exhibited high ALC nadir levels, while the remaining 110 (58.2%) exhibited low ALC nadir levels.

### ALC Nadir During RT Is Associated With Survival Outcomes

**Figure 2:**
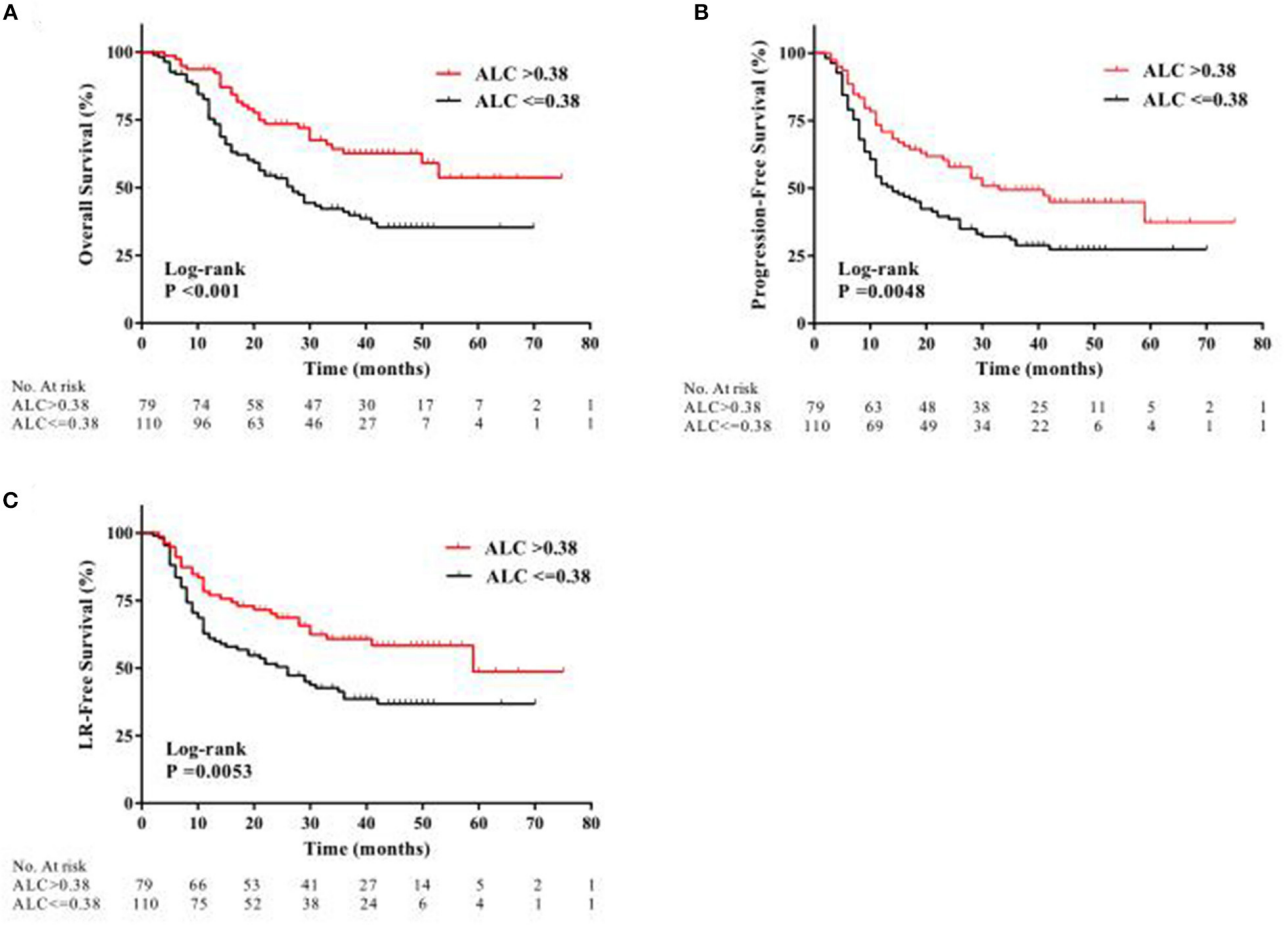
Kaplan-Meier curves showing patient clinical outcomes: **(A)** overall survival, **(B)** progression-free survival, and **(C)** local recurrence-free (LR) survival between patients with high ALC nadir (red line) and with low ALC nadir (black line) during radiotherapy.

At the median follow-up time of 46 months, 95 patients (50.3%) had died. Compared with patients with high ALC nadir, patients with low ALC nadir had a significantly worse OS [hazard ratio [HR], 2.08; 95% confidence interval [CI], 1.37–3.05; *P* < 0.001; Figure 2A]. After adjusting for risk factors, multivariate analysis (MVA) revealed that tumor localization in the middle or lower third of the esophagus (HR, 2.94; 95% CI, 1.88–4.60; *P* < 0.001), stage III or IV tumor (HR, 2.34; 95% CI, 1.29–4.22; *P* = 0.005), treatment with RT alone (HR, 1.58; 95% CI, 1.03–2.43; *P* = 0.037), and low ALC nadir during RT (HR, 1.87; 95% CI, 1.20–2.94; *P* = 0.006) were independent factor predicting unfavorable OS (Table 2).

**Table 2:** Univariate and multivariate Cox regression analysis of factors associated with clinical outcomes.

**Characteristics**	**OS**				**PFS**					**LRFS**					
	**Univariate analysis**		**Multivariate analysis**		**Univariate analysis**		**Multivariate analysis**			**Univariate analysis**			**Multivariate analysis**		
	**HR (95% CI)**	***P*-value**	**HR (95% CI)**	***P*-value**	**HR (95% CI)**	***P*-value**	**HR**	**95% CI**	***P-*value**	**HR**	**95% CI**	***P*-value**	**HR**	**95% CI**	***P*-value**
Age															
≤67	1				1					1					
>67	1.62(1.08–2.44)	**0.020**		NS	1.13 (0.79–1.62)	0.493				1.33 (0.89–1.99)		0.169			
Gender															
Male	1				1										
Female	0.77 (0.47–1.25)	0.292			0.81 (0.53–1.23)	0.321				0.75 (0.46–1.22)		0.250			
Smoking															
Ever	1				1					1			1		
Never	0.75 (0.50–1.13)	0.164			0.78 (0.55–1.12)	0.184				1.77 (1.17–2.67)		**0.007**	1.66 (1.09–2.50)		**0.017**
Drinking															
Ever	1				1					1					
Never	0.63 (0.42–0.95)	**0.027**		NS	0.64 (0.45–0.92)	**0.015**			NS	1.73 (1.16–2.60)		**0.008**			NS
Tumor location															
Cervical or Upper	1		1		1		1			1			1		
Mid or Lower	3.02 (1.96–4.64)	**<0.001**	2.94 (1.88–4.60)	**<0.001**	2.33 (1.61–3.37)	**<0.001**	2.59 (1.78–3.76)		**<0.001**	2.61 (1.70–3.99)		**<0.001**	2.64 (1.72–4.04)		**<0.001**
Tumor length															
≤4.4 cm	1				1					1					
>4.4 cm	1.86 (1.23–2.80)	**0.003**		NS	1.70 (1.18–2.45)	**0.004**			NS	1.51 (1.01–2.28)		**0.047**			NS
Clinical stage															
I–II	1		1		1		1			1					
III–IVA	2.07 (1.17–3.66)	**0.012**	2.34 (1.29–4.22)	**0.005**	1.76 (1.09–2.85)	**0.021**	1.84 (1.11–3.04)		**0.018**	1.45 (0.87–2.43)		0.156			
Treatment regimen															
RT combined with chemo	1		1		1					1					
RT alone	1.73 (1.15–2.59)	**0.009**	1.58 (1.03–2.43)	**0.037**	1.24 (0.87–1.78)	0.233				1.55 (1.03–2.32)		**0.035**			NS
Chemo regimen															
Platinum/taxane	1				1					1					
Platinum/5-FU	1.36 (0.52–3.56)	0.531			1.13 (0.57–2.26)	0.723				1.93 (0.80–4.65)		0.145			
Other	1.58 (0.98–2.54)	0.058			1.16 (0.79–1.71)	0.441				1.33 (0.80–2.21)		0.276			
RT technology															
3D-CRT	1				1					1					
IMRT	0.76 (0.50–1.14)	0.184			0.67 (0.46–0.96)	**0.030**			NS	0.71 (0.47–1.08)		0.106			
RT dose															
≤60 Gy	1				1					1					
>60 Gy	0.73 (0.44–1.22)	0.233			0.77 (0.49–1.21)	0.257				0.73 (0.44–1.22)		0.231			
Pre-treatment ALC															
≤1.73 × 10^3^ cells/μL	1				1					1					
>1.73 × 10^3^ cells/μL	0.87 (0.58–1.31)	0.507			0.78 (0.55–1.12)	0.180				0.66 (0.44–1.00)		0.050			
ALC nadir during RT															
>0.38 × 10^3^ cells/μL	1		1		1		1			1			1		
≤0.38 × 10^3^ cells/μL	2.10 (1.35–3.26)	**0.001**	1.87 (1.20–2.94)	**0.006**	1.69 (1.16–2.47)	**0.006**	1.55 (1.05–2.29)		**0.028**	1.82 (1.18–2.80)		**0.007**	1.91 (1.23–2.94)		**0.004**

*OS, overall survival; PFS, progression-free survival; LRFS, local recurrence-free survival;HR, hazard ratio; CI, confidence interval; RT, radiotherapy; chemo, chemotherapy; 5-FU, 5-fluorouracil; IMRT, intensity modulated radiotherapy; 3D-CRT, three-dimensional conformal radiotherapy; ALC, absolute lymphocyte count; NS, non-significant. Bold values indicate a statistically difference in statistical analysis (P < 0.05)*.

During follow-up, a total of 120 patients (63.5%) had locoregional or distant progression, with a median PFS of 19 months. Kaplan-Meier analysis indicated a significantly worse PFS in patients with low ALC nadir compared with patients with high ALC nadir (HR, 1.69; 95% CI, 1.18–2.43; *P* = 0.0048; Figure 2B). According to UVA, drinking history, tumor localization, tumor size, clinical stage, RT method, and ALC nadir, were all significantly associated with PFS (all *P* < 0.05; Table 2). MVA revealed that tumor localization in the middle or lower third of the esophagus (HR, 2.58; 95% CI, 1.78–3.76; *P* < 0.001), stage III or IV tumor (HR, 1.84; 95% CI, 1.11–3.04; *P* = 0.018), and low ALC nadir (HR, 1.55; 95% CI, 1.05–2.29; *P* = 0.028) during RT were significantly associated with inferior PFS (Table 2).

Locoregional progression was observed in 94 patients (49.7%) during follow-up. Similarly, low ALC nadir during RT predicted for worse LRFS (HR, 1.81; 95% CI, 1.20–2.70; *P* = 0.0053; Figure 2C). Furthermore, MVA analysis showed that smoking history (HR, 1.66; 95% CI, 1.09–2.50; *P* = 0.017), tumor localization in the middle or lower third of the esophagus (HR, 2.64; 95% CI, 1.72–4.04; *P* < 0.001), and low ALC nadir during RT (HR, 1.91; 95% CI, 1.23–2.94; *P* = 0.004) were significantly associated with worse LRFS (Table 2).

### Predictors of Low ALC Nadir During RT

One hundred ten patients (58.2%) exhibited low ALC nadir during RT. We found no significant differences in age, smoking history, drinking history, tumor localization, treatment regimen, RT method, RT dose, and pre-treatment ALC values between patients with low and high ALC nadir. Pearson's chi-squared tests indicated that male patients (*P* = 0.044), patients with larger tumors (>4.4 cm; *P* = 0.006), and advanced clinical stage patients (*P* < 0.001) were more likely to have a low ALC nadir during RT (Table 1).

**Figure 3:**
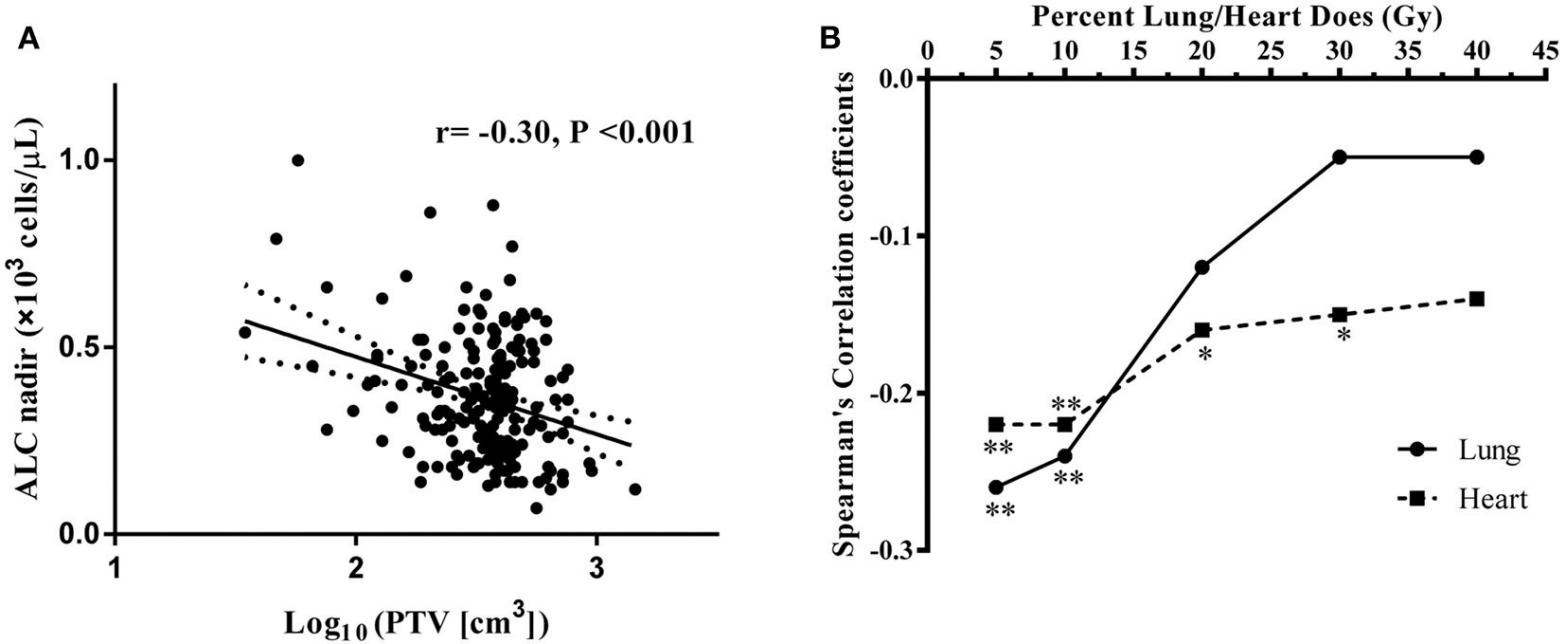
**(A)** Correlation between log_10_(PTV) and absolute lymphocyte count (ALC) nadir during RT. **(B)** Spearman correlation coefficients between the percentages of lung/heart dose (Gy) and lymphocyte nadirs at varying percentages of lung (solid line)/heart doses (dashed line) for patient. Significance indicated at **P < 0.01, and *P < 0.05.

The median PTV was significantly higher in patients with low ALC nadir (418.5 ± 204.7 cm^3^) compared with patients with high ALC nadir (347.7 ± 157.4 cm^3^; *P* = 0.023). Consistently, a significant negative correlation was observed between ALC nadir during RT and log_10_(PTV) (*r* = −0.30; *P* < 0.001; Figure 3A). To examine this further, we evaluated the correlation of low ALC nadir with lung or heart dosimetry parameters. To this end, we assessed the Spearman correlation between low ALC nadir and the percentage of lung and heart receiving 5 to 40 Gy (lung V5–V40, heart V5–V40; Figure 3B). We found that high lung V5 (*r* = −0.26; *P* < 0.001), lung V10 (*r* = −0.24; *P* = 0.001), heart V5 (*r* = −0.22; *P* = 0.002), and heart V10 (*r* = −0.22; *P* = 0.002) strongly correlated with lower ALC nadir (*P* < 0.01). This correlation subsequently lessened at heart V20 (*r* = −0.16; *P* = 0.029) and heart V30 (*r* = −0.15; *P* = 0.037). Lung V20–V40, as well as heart V40, showed no significant correlation with low ALC nadir (*P* ≥ 0.05). Stepwise multivariate logistic regression demonstrated that patients with tumor stage III-IVA [odds ratio [OR], 3.46; 95% CI, 1.56–7.68; *P* = 0.002], lower ALC value before treatment (OR, 0.50; 95% CI, 0.27–0.93; *P* = 0.028), larger Log_10_(PTV) (OR, 8.31; 95% CI, 1.65–41.78; *P* = 0.01), higher heart V10 (OR, 1.05; 95% CI, 1.02–1.09; *P* = 0.003), and higher heart V20 (OR, 0.95; 95% CI, 0.91–1.00; *P* = 0.028) had a lower ALC nadir (Table 3). Of note, although patients were treated with different therapeutic schedules, there were no significant effects of therapeutic scheduling on the ALC nadir level.

**Table 3:** Univariate and multivariate logistic regression of factors associated with low ALC nadir.

**Characteristics**	**Univariate analysis**		**Multivariate analysis**	
	**OR (95% CI)**	***p*-value**	**OR (95% CI)**	***p*-value**
Age (years)^*^	0.99 (0.96–1.02)	0.586		
Tumor location				
Cervical or Upper	1			
Mid or Lower	1.20 (0.68–2.15)	0.531		
Tumor length (cm)^*^	1.28 (1.09–1.49)	**0.003**		NS
Clinical stage				
I–II	1		1	
III–IVA	3.56 (1.72–7.38)	**0.001**	3.46 (1.56–7.68)	**0.002**
Treatment regimen				
RT combined with chemo	1			
RT alone	0.97 (0.54–1.73)	0.910		
Chemo regimen				
Platinum/taxane	1			
Platinum/5-FU	2.31 (0.73–7.28)	0.154		
Other	0.88 (0.47–1.63)	0.678		
RT technology				
3D-CRT	1			
IMRT	1.52 (0.83–2.79)	0.173		
RT dose (Gy)^*^	1.00 (0.93–1.09)	0.844		
Pre-treatment ALC (×10^3^ cells/μL)^*^	0.56 (0.32–0.97)	**0.039**	0.50 (0.27–0.93)	**0.028**
Log_10_ [PTV [cm^3^]]^*^	5.34 (1.41–20.16)	**0.013**	8.31 (1.65–41.78)	**0.010**
Lung DVH^*^				
V5 (%)	1.03 (1.01–1.06)	**0.001**		NS
V10 (%)	1.04 (1.01–1.07)	**0.004**		NS
V20 (%)	1.05 (1.00–1.10)	**0.034**		NS
V30 (%)	1.06 (0.98–1.14)	0.186		NS
V40 (%)	1.08 (0.96–1.23)	0.213		NS
Mean does (Gy)	1.17 (1.05–1.30)	**0.004**		NS
Heart DVH^*^				
V5 (%)	1.01 (1.00–1.02)	**0.006**		NS
V10 (%)	1.01 (1.00–1.02)	**0.005**	1.05 (1.01–1.09)	**0.003**
V20 (%)	1.01 (1.00–1.03)	0.073	0.95 (0.91–1.00)	**0.028**
V30 (%)	1.01 (0.99–1.03)	0.251		NS
V40 (%)	1.01 (0.98–1.05)	0.461		NS
Mean does (Gy)	1.05 (1.01–1.09)	**0.007**		NS

*ALC, absolute lymphocyte count; OR, odds ratio; CI, confidence interval; RT, radiotherapy; chemo, chemotherapy; 5-FU, 5-fluorouracil; 3D-CRT, three-dimensional conformal radiotherapy; IMRT, intensity modulated radiotherapy; PTV, planning target volume; DVH, dose-volume histogram; Lung V5/10/20/30/40, the percentage of lung receiving 5/10/20/30/40 Gy; Heart V5/10/20/30/40, the percentage of heart receiving 5/10/20/30/40 Gy; NS, non-significant*.

* *Indicates a continuous variable with units indicated in parenthesis. Bold values indicate a statistically difference in statistical analysis (P < 0.05)*.

## Discussion

In this study, we observed a significant lymphocyte depletion following RT in ESCC patients, followed by a gradual recovery in ALC after the end of RT. We also found that a lower ALC nadir during RT was significantly associated with poor OS, PFS, and LRFS. The prognostic value of ALC nadir during RT remained significant after adjustment for confounding risk factors. Finally, we found that low ALC nadir was associated with the clinical stage, ALC before treatment, PTV, heart V10, and heart V20. These results are very clinically relevant, because with the development of modern RT techniques, RIL has emerged as a strong and potentially modifiable risk factor. Adjusting RT parameters to minimize immunosuppression caused by RIL may help optimize the therapeutic effect of RT in ESCC and other malignancies.

Our study indicated that ALC value was a prognostic factor in patients with ESCC who received definitive RT. Lymphocytes play critical roles in promoting systemic anti-tumor immune responses. Lymphopenia is a main manifestation of immunosuppression, and several retrospective studies have demonstrated a strong link between treatment-induced lymphopenia and inferior outcomes in a wide variety of cancers, including glioblastoma, lung cancer, pancreatic cancer, cervical cancer, and EC (9–14). Furthermore, a prospective study has also confirmed the correlation between lymphocyte count and outcomes in patients with high-grade glioma (16). However, the definition of treatment-related lymphopenia varied among these studies. Previous studies have also reported that maintaining a high ALC during treatment was associated with higher pathologic complete response (pCR) rates in patients with rectal cancer, gastric cancer, and EC (17–19). In our study, we used clinical CR rates as state variables to determine the optimal cut-off value of ALC nadir during RT. Consistent with previously reported findings, our data indicated that a high ALC nadir during RT was associated with higher CR rates and improved survival outcomes. Interestingly, some retrospective studies have reported that low ALC values before treatment are associated with poor outcomes in cancer patients (20, 21). Our results showed that low ALC nadir during RT was associated with low ALC value before treatment. Given the exquisite radiosensitivity of lymphocytes, a change in lymphocyte count may also be a prognostic factor worthy of attention.

By MVA, we identified several radiation treatment parameters that could significantly predict low ALC nadir during RT. Lymphocytes are the most radiosensitive hematopoietic cells and are frequently depleted by RT using a 50% lethal dose of 1 to 2 Gy (5, 6). Nevertheless, the mechanisms responsible for RIL remain unclear. Lymphoid organ radiation, including bone marrow, thymus, spleen, and lymph nodes, could likely contribute to RIL (22–24). Radiation can directly damage hematopoietic stem cells in bone marrow or mature lymphocytes in lymphoid tissues, as well as stimulate the secretion of immunosuppressive cytokines, promoting immunosuppression (25). Additionally, as EC develops in close proximity to the heart and lungs, lymphocytes receive a significant dose of radiation through the large blood vessels that are in the radiation field (14). Tang et al. (12) analyzed the relationship between DVH parameters and lymphocyte nadir in 771 non-small cell lung cancer (NSCLC) patients and observed that lymphocyte nadir was associated with gross tumor volume (GTV). Rudra et al. (26) assessed the effects of different RT volumes on lymphopenia risk in glioblastoma patients and found that the reduction in the irradiated brain volume might mitigate treatment-induced lymphopenia. Furthermore, the results of RTOG 0617 showed that higher cardiac doses and larger PTV were significant predictors of poor OS (27). Due to the esophagus's anatomical position near the heart, lungs, and several large blood vessels, we can reasonably extrapolate that these effects would be enhanced in EC treated with RT. Our study supported that PTV, heart V10, and heart V20 are significant prognostic factors of low ALC nadir. Our findings also suggested that larger volumes of radiation result in more severe damage in lymphocytes. Analysis of DVH parameters showed that lung V5–V10 and heart V5–V10 had the highest correlation coefficients, suggesting that RIL occurs primarily as a result of frequent low-dose radiation damage in circulating lymphocytes. In addition, we found that the administration of chemotherapy, in combination with RT, had no significant effect on lymphocytes.

In addition to immunosuppressive effects, immunostimulating roles have also been described for RT. RT exerts strong anti-tumor effects by increasing the immunogenicity of cancer cells (28). Furthermore, the RT-induced modulation of the tumor microenvironment can promote the recruitment of immune cells in the tumor, as well as enhance the tumor cell recognition and elimination by immune cells (29). Preclinical studies demonstrated that PD-L1 expression was upregulated in the tumor microenvironment after RT (30, 31). Since the approval of anti-CTLA4 therapy (ipilimumab) for unresectable or metastatic melanoma in 2011 (32), the development of anti-cancer immunotherapy agents has progressed rapidly. As the number of preclinical and clinical studies assessing the anti-tumor effects of RT combined with immunotherapy, it is vital to eliminate the immunosuppressive effects of RT to achieve optimal anti-cancer effects (33). In clinical practice, however, we have traditionally ignored the effect of radiation parameters on lymphocytes in the development of RT plans. Our findings suggested that reducing radiation field size could minimize the risk of RIL during RT. The radiation target volume of EC has remained a topic of persistent controversy among radiation oncologists. One of the most controversial points is whether to opt for elective nodal irradiation (ENI) or involved-field irradiation (IFI). ENI involves delivering RT to the primary tumor, as well as the irradiation of clinically uninvolved regional lymph nodes at risk of micrometastases of the treated disease (34). However, previous studies confirmed that ENI did not change failure patterns and survival outcomes of local advanced stage EC after definitive chemoradiotheraty (35–38). Compared with ENI, IFI has a smaller radiation target volume. Combined with the results of our study, IFI may be a better choice for reducing RIL. Further research is required to test this hypothesis. Additionally, the ability of proton beam therapy (PBT) to conform high radiation dose to the tumor volume while reducing the unintentional radiation dose to adjacent healthy tissues has the potential to decrease RIL (39, 40). RT could quite possibly be an immunologic adjuvant if the RT plan is right.

Our study has several limitations. Importantly, our study is a single institution retrospective study; hence, there was a significant risk of selection bias and information bias. Although in our institution, we perform routine blood tests before RT and weekly during RT, there is considerable variation in this procedure. For patients with missing blood data at a time point of interest, the closest value to the desired date was used. Moreover, there were several potential confounding variables in our study, such as auxiliary medications or infections that might have affected the lymphocyte count. Although all the patients of our study received definitive RT, there might have been variation in the therapeutic scheduling due to the wide time span of treatment. Finally, even though using ROC curve analysis, we identified the cut-off ALC nadir value that most accurately predicts response, the optimal threshold needs to be validated in larger cohorts from multi-institutional studies.

Despite these limitations, our study suggests a strong link between RIL and prognosis in patients with ESCC. It also identifies several parameters that could modulate the lymphocyte count during RT, providing further insight into the mechanisms behind RIL. Additionally, our findings further demonstrate the importance of maintaining an intact immune system during anti-tumor therapy. Adjusting potential risk factors of RIL to enhance host immunity may help optimize the therapeutic benefit of RT in patients with ESCC and other malignancies.

## Data Availability Statement

The raw data supporting the conclusions of this article will be made available by the authors, without undue reservation.

## Ethics Statement

The studies involving human participants were reviewed and approved by Shandong Cancer Hospital and Institute, Shandong First Medical University and Shandong Academy of Medical Sciences. The patients/participants provided their written informed consent to participate in this study. Written informed consent was obtained from the individual(s) for the publication of any potentially identifiable images or data included in this article.

## Author Contributions

ML and XW designed the study. XW drafted the manuscript. ZZ and PW participated in data collection. XG and LZ coordinated, edited, and finalized the drafting of the manuscript. All authors read and approved the final manuscript.

## Conflict of Interest

The authors declare that the research was conducted in the absence of any commercial or financial relationships that could be construed as a potential conflict of interest.

## Abbreviations

ALC: absolute lymphocyte count
AJCC: American Joint Committee on Cancer
CI: confidence interval
CR: complete response
CRT: chemoradiation therapy
CT: computed tomography
dCRT: definitive chemoradiotherapy
DVH: dose-volume histogram
EAC: esophageal adenocarcinoma
ECOG: Eastern Cooperative Oncology Group
ESCC: esophageal squamous cell carcinoma
ENI: elective nodal irradiation
EUS: endoscopic ultrasonography
GTV: gross tumor volume
HR: hazard ratio
IFI: involved-field irradiation
IMRT: intensity modulated radiotherapy
LRFS: local recurrence-free survival
MVA: multivariate analysis
NSCLC: non-small cell lung cancer
OARs: organs-at-risk
OR: odds ratio
OS: overall survival
PBT: proton beam therapy
pCR: pathologic complete response
PET: positron emission tomography
PFS: progression-free survival
PTV: Planning target volume
RECIST: Response Evaluation Criteria in Solid Tumors
RIL: radiation-induced lymphopenia
ROC: receiver operating characteristic
RT: radiotherapy
RTOG: Radiation Therapy Oncology Group
UVA: univariate analysis
3D-CRT: three-dimensional conformal radiation therapy
5-FU: 5-fluorouracil.
