# Corrigendum: Low Lymphocyte Count Is Associated With Radiotherapy Parameters and Affects the Outcomes of Esophageal Squamous Cell Carcinoma Patients

**DOI:** 10.3389/fonc.2020.630877

**Published:** 2021-01-12

**Authors:** Xin Wang, Zongxing Zhao, Peiliang Wang, Xiaotao Geng, Liqiong Zhu, Minghuan Li

**Affiliations:** ^1^ Department of Clinical Medicine, Shandong First Medical University and Shandong Academy of Medical Sciences, Jinan, China; ^2^ Department of Radiation Oncology, Shandong Cancer Hospital and Institute, Shandong First Medical University and Shandong Academy of Medical Sciences, Jinan, China; ^3^ Department of Radiation Oncology, Liaocheng People’s Hospital, Liaocheng, China; ^4^ School of Medicine, Shandong University, Jinan, China

**Keywords:** radiotherapy, esophageal squamous cell carcinoma, radiation-induced lymphopenia, prognosis, immunosuppression

In the original article, there was a mistake in **Figure 2** as published. Due to the authors’ carelessness, the line colors for “high ALC nadir” group and “low ALC nadir” group in **Figure 2** were mistakenly reversed. The corrected **Figure 2** and its legend appear below.

**Figure 2:**
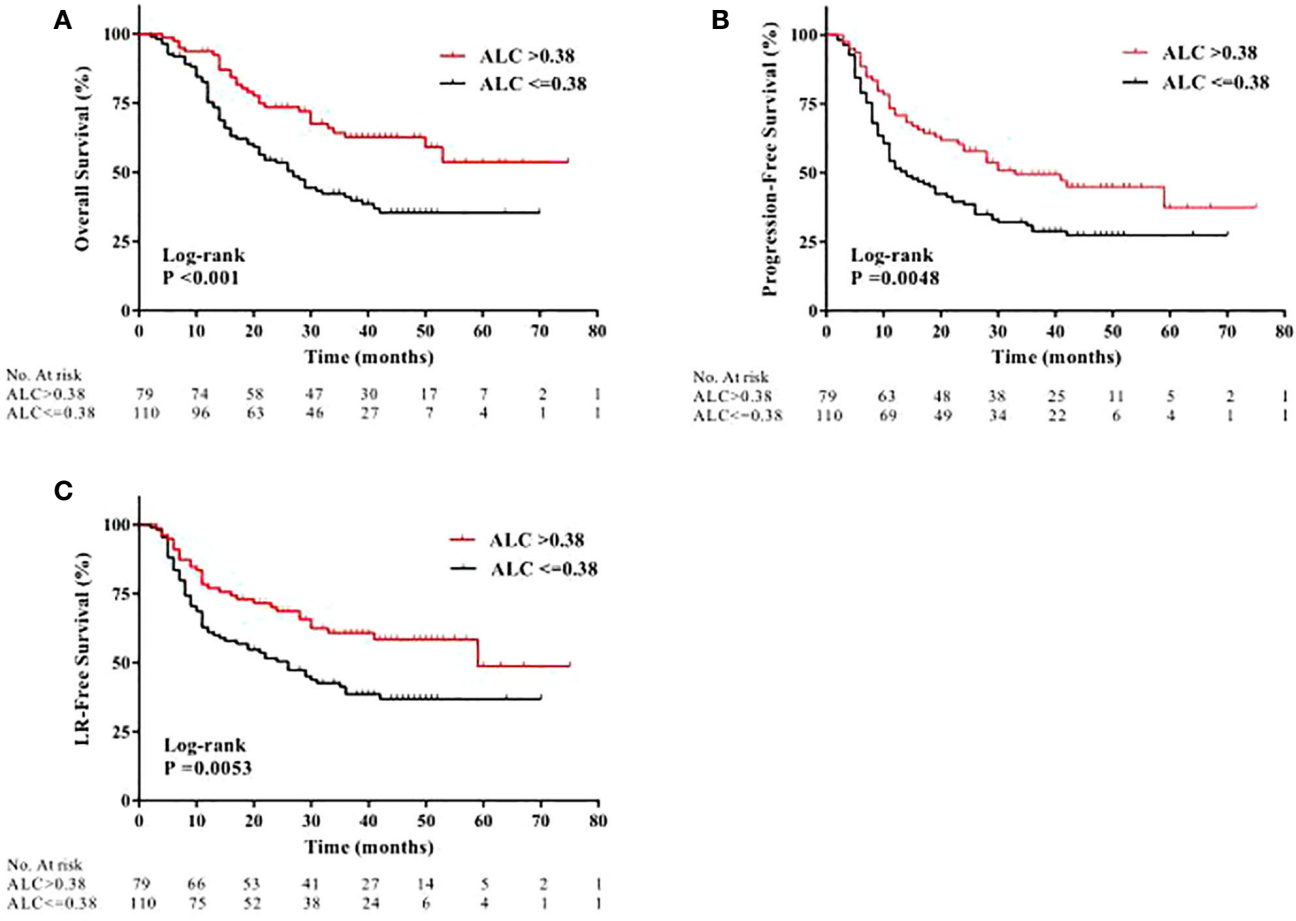
Kaplan-Meier curves showing patient clinical outcomes: **(A)** overall survival, **(B)** progression-free survival, and **(C)** local recurrence-free (LR) survival between patients with high ALC nadir (red line) and with low ALC nadir (black line) during radiotherapy.

The authors apologize for this error and state that this does not change the scientific conclusions of the article in any way. The original article has been updated.

